# Lovastatin Alleviates Endothelial-to-Mesenchymal Transition in Glomeruli via Suppression of Oxidative Stress and TGF-β1 Signaling

**DOI:** 10.3389/fphar.2017.00473

**Published:** 2017-07-18

**Authors:** Zejun Ma, Lili Zhu, Yan Liu, Zhida Wang, Yang Yang, Liming Chen, Qiulun Lu

**Affiliations:** ^1^Key Laboratory of Hormones and Development (Ministry of Health), Tianjin Key Laboratory of Metabolic Diseases, Tianjin Metabolic Diseases Hospital and Tianjin Institute of Endocrinology, Tianjin Medical University Tianjin, China; ^2^Tianjin Medical Devices Quality Supervision and Testing Center Tianjin, China; ^3^Key Laboratory of Molecular Biophysics of the Ministry of Education, College of Life Science and Technology and Center for Human Genome Research, Huazhong University of Science and Technology Wuhan, China

**Keywords:** diabetic nephropathy, endothelial-to-mesenchymal transition, renal fibrosis, oxidative stress, lovastatin

## Abstract

Statins may decrease chronic kidney diseases (CKDs) risk, but their underlying molecular mechanisms are not completely understood. Recent studies indicate Endothelial-to-mesenchymal transition (EndMT) plays an important role contributing to renal interstitial fibrosis. In the present study, we first investigated whether lovastatin could ameliorate renal fibrosis via suppression of EndMT and its possible mechanism. *In vitro* experiments, lovastatin significantly ameliorated microalbuminuria and pathologic changes in diabetic rats. Double labeling immunofluorescence showed lovastatin could inhibit EndMT in glomeruli. Furthermore, lovastatin could inhibit oxidative stress and down-regulate TGF-β1-Smad signaling. Consistent alterations were observed *in vivo* that lovastatin substantially suppressed EndMT and TGF-β1 signaling induced by high glucose in glomerular endothelial cells (GEnCs). These data indicated that lovastatin could ameliorate EndMT in glomeruli in diabetic nephropathy, the mechanism of which might be at least partly through suppression of oxidative stress and TGF-β1/Smad signaling pathway.

## Introduction

Diabetic nephropathy (DN) has become the primary microvascular complication of diabetes and may lead to end-stage renal disease (ESRD) (Packham et al., 2012; Tomino and Gohda, 2015). DN is caused by a complex interplay of factors, such as hyperglycemia, hypertension, oxidative stress, chemokines, and inflammation (Elmarakby and Sullivan, 2012; Bhattacharjee et al., 2016). Till now, the treatment for DN is very limited. Once DN progresses to ESRD, the cost of management is very high and is associated with an increased cardiovascular mortality (Palmer Alves and Lewis, 2010; Xue et al., 2017). Therefore, the search for the novel therapeutic drug to delay the progression of DN is essential.

Early feature of DN is hyperfiltration in glomerulus and appearance of microalbuminuria (Fu et al., 2015). Previous studies mainly focused on the podocytes, which were regarded as the main site of the filtration membrane to maintain the glomerular filtration barrier (Mathieson, 2009; Nagata, 2016). However, increasing evidence suggests that other structures of the glomerular area also play a key role (Quaggin and Kreidberg, 2008; Scott and Quaggin, 2015). Among them, GEnCs are now increasingly regarded to play a significant role in maintaining the glomerular filtration barrier (Satchell, 2012; Fu et al., 2015). GEnCs impairment may result in increased filtration of albumin and developmental and pathological processes of renal fibrosis. Recently, EndMT has emerged as a potentially important mechanism involved in glomerular endothelial impairment (Peng et al., 2016; Zhao et al., 2016). EndMT is a process in which endothelial cells (ECs) loss the endothelial phenotype and gain mesenchymal feature such as vimentin and α-smooth muscle actin (α-SMA) (Liang et al., 2016). In addition, suppression of EndMT could regain the endothelial phenotype and avoid organ fibrosis (Zeisberg et al., 2007; Chen et al., 2016; Deng et al., 2016).

Statins, inhibitor of 3-hydroxy-3-methylglutaryl-coenzyme A reductase, can inhibit cholesterol synthesis and are prescribed to cope with endogenous hypercholesterolemia (Aronow, 2017). Recent studies demonstrated that statins have emerged as novel renoprotective drugs independently of their lipid-lowering effect in a variety of glomerular diseases including diabetic nephropathy (Epstein and Campese, 2005; Kolavennu et al., 2008; Peng et al., 2013; Shen et al., 2016), whereas the underlying mechanism has not been understood completely. In the present study, we evaluated whether lovastatin could attenuate renal fibrosis in diabetic rats via suppression of EndMT and the possible mechanisms behind.

## Methods

### Animal model and experimental protocol

All the experimental protocols that involve animals were approved by the Animal Care and Use Committee of Tianjin Medical University. All rats were housed in cages under a 12:12-h light: dark cycle with *ad libitum* access to food and water. 30 male Sprague-Dawley rats (8-week old) weighing 150–200 g were separated into three groups: Normal control group (NC, *n* = 10); (2) diabetic group (DM, *n* = 10); (3) lovastatin-treated diabetic group (DM+ lovastatin, *n* = 10). DM was induced by intraperitoneal injection with STZ 150 mg/kg (Sigma, St. Louis, MO, USA) as described previously (Tesch and Allen, 2007). The blood glucose >16 mmol/L was regarded as diabetic. For the lovastatin-treated diabetic group, these rats were treated with oral lovastatin (5 mg/kg/day) in distilled water. Rats in NC and DM groups were respectively administered orally with an equal volume of distilled water. After 16-week treatment, animals were kept in metabolic cages to collect 24 h urine to measure urinary microalbuminuria. Blood samples were collected from the abdominal aorta for measuring the biochemical parameter. BUN and Scr concentrations were detected with an Olympus 400 clinical chemistry analyzer. The left kidney was removed and weighed to calculate the kidney weight to body weight (KW/BW) ratio. The left kidney was kept in neutral-buffered formalin (10%) for morphological and immunohistochemical analysis. The right kidney was frozen for protein extraction, RNA preparation and Immunofluorescence.

### Cell culture and treatments

GEnCs were gained from Cell Biologics. GEnCs were cultured in endothelial cell medium (ECM) containing 10% fetal bovine serum and 1% endothelial cell growth supplement. The cells were cultivated in a humidified atmosphere at 37°C of 5% CO2. To observe the effect of lovastatin, cells were divided into: 192 normal control group (NG group): 5.6 mmol/L glucose, 193 osmotic pressure control group (OP group): 5.6 mmol/L glucose + 24.4 mmol/L mannitol, 194 high glucose group (HG group): 30 mmol/L glucose, 195 high glucose + different concentrations of lovastatin group (HG + lovastatin 1 μM group, HG + lovastatin 5 μM group and HG + lovastatin 10 μM group, respectively). After incubation with different concentrations of glucose for 48 h, condition media were collected and cells were harvested for further analysis.

### HE staining and masson staining of renal tissues

The renal tissue was preserved in 10% formaldehyde and then embedded in paraffin. 4 μm thick sections were used for HE staining and Masson staining. After staining, the pathological changes of glomeruli were evaluated at × 400 magnification by two investigators in a blinded manner under an IDA-2,000 high-resolution digital microscope and image analysis system.

### Immunohistochemistry staining

Immunohistochemistry was performed as previously described (Brenneman et al., 2016). In brief, after dewaxed in xylene and dehydrated in graded ethanol, the renal tissue slides (4 μm thick) were microwaved in citrate buffer for antigen retrieval and blocked with 3% H_2_O_2_ for 10 min. Then the renal tissue were incubated with the following primary antibodies: anti-NOX-4 (1:100), anti-TGF-β1 (1:100), anti-fibronectin (1:100), anti-collagen IV (1:100), anti-VE-cadherin (1:200 dilution), and anti-vimentin (1:200 dilution). After rinsing three times in PBS and incubated for 45 min with second antibodies; the sections were visualized with a Diaminobenzidine (DAB) kit and counterstained with Haematoxylin. Then the slides were checked in 10 randomly selected cortical sections at a magnification × 400. The mean light density of each visual field was taken to calculate the number and percentage of the positive area of the Glomeruli area.

### Real-time quantitative PCR

Total RNA was isolated from kidney or cells and then reverse-transcribed into cDNA. The real-time RT-PCR quantitation was carried out on the CFX96 real-time PCR system (Bio-Rad, USA) using a TaKaRa SYBR Green PCR kit (SYBR PremixEx TaqTM II, Takara, Japan) as described previously (Ma et al., 2015). The PCR primer sequences used in this study were as follows:

Collagen IV Forward: 5′-CCATCTGTGGACCATGGCTT-3′Reverse: 5′-GCGAAGTTGCAGACGTTGTT -3′.

TGF-β1 Forward: 5′-GTTTCCGTGTCCTCTTCCCA-3′ Reverse: 5′-GGACAGGGCTGGTTCATAAAT-3′.

Fibronectin Forward: 5′-CTTGCGGGCACCAGACCT-3′ Reverse: 5′-CTTCATCCGAGTGTCTGTCT-3′.

GAPDH Forward: 5′-GGCACAGTCAAGGCTGAGAATG-3′ Reverse: 5′-ATGGTGGTGAAGACGCCAGTA-3′.

Experiments were performed in triplicate. The Relative mRNA changes were calculated using 2^−ΔΔCT^ analysis.

### Western blotting

Western blots were performed as described previously (Ma et al., 2015). Proteins samples from kidney or cell were isolated and equal amounts of protein were loaded to 10 or 12% SDS-PAGE gels and then transferred to PVDF membranes. The membranes were incubated with primary antibodies against TGF-β1 (Santa Cruz Biotechnology, 1:1000 dilution), fibronectin (Abcam, 1:1,000 dilution), collagen IV (Abcam, 1:1,500 dilution), VE-cadherin (Cell Signaling Technology, 1:1,000 dilution), CD31 (Abcam, 1:1,000 dilution), α-SMA(Thermo Fisher Scientific, 1:1,000 dilution), vimentin (Santa Cruz Biotechnology, 1:2,000 dilution), phospho-Smad2 (Cell Signaling Technology, 1:1,000 dilution), Smad2 (Cell Signaling Technology, 1:1,000 dilution), phospho-Smad3 (Cell Signaling Technology, 1:1,000 dilution), Smad3 (Cell Signaling Technology, 1:1,000 dilution), NOX-4 (Santa Cruz Biotechnology, 1:1,000 dilution), or GAPDH (Santa Cruz Biotechnology, 1:1,000 dilution) overnight at 4°C, then incubated with secondary antibody for 1 h. ECL chromogenic substrates were applied on the membrane for development and exposure in dark room. GAPDH served as an internal control for calculating relative protein concentration.

### Immunofluorescence

Frozen sections of kidney tissues were fixed in pre-cooled acetone for 10 min, and then kidney tissues were blocked with 5% normal bovine serum. The kidney tissues were incubated with primary antibodies (rat anti-CD31 1:200, mouse anti-α-SMA 1:200) overnight at 4°C. After washed with PBS, the kidney tissues were incubated with secondary antibodies (Alexa Fluor 488 goat anti-rat 1-1:200; Alexa Fluor 549 anti-mouse 1:200), then counterstained with DAPI. Ten visual fields per kidney were examined in a blinded manner for co-localization of endothelial and fibroblast markers.

### DHE staining in kidney sections and measurement of SOD and malondialdehyde (MDA) levels

In this study, superoxide production in the kidney was detected by dihydroethidium (DHE) staining. Frozen kidney sections in OCT compound (5 μm thick) for 24 h were stained with DHE (10 μmol/l) for 30 min at room temperature protected from light. Red staining representing oxidative stress was measured with Image Pro Plus software. SOD activity and MDA content in kidney were measured with commercial kits according to the manufacturers' protocols.

### Statistical analysis

The data were presented as mean ± standard deviation (*SD*). Statistical analyses were carried out by one-way analysis of variance (ANOVA) or the LSD *t*-test. The data were assessed with GraphPad Prism version 5.0 (GraphPad Prism, San Diego, CA, USA). *P*-value < 0.05 were considered to be statistically significant.

## Results

### *In vitro* experiments

#### Effects of lovastatin on metabolic parameters

**Figure 1:**
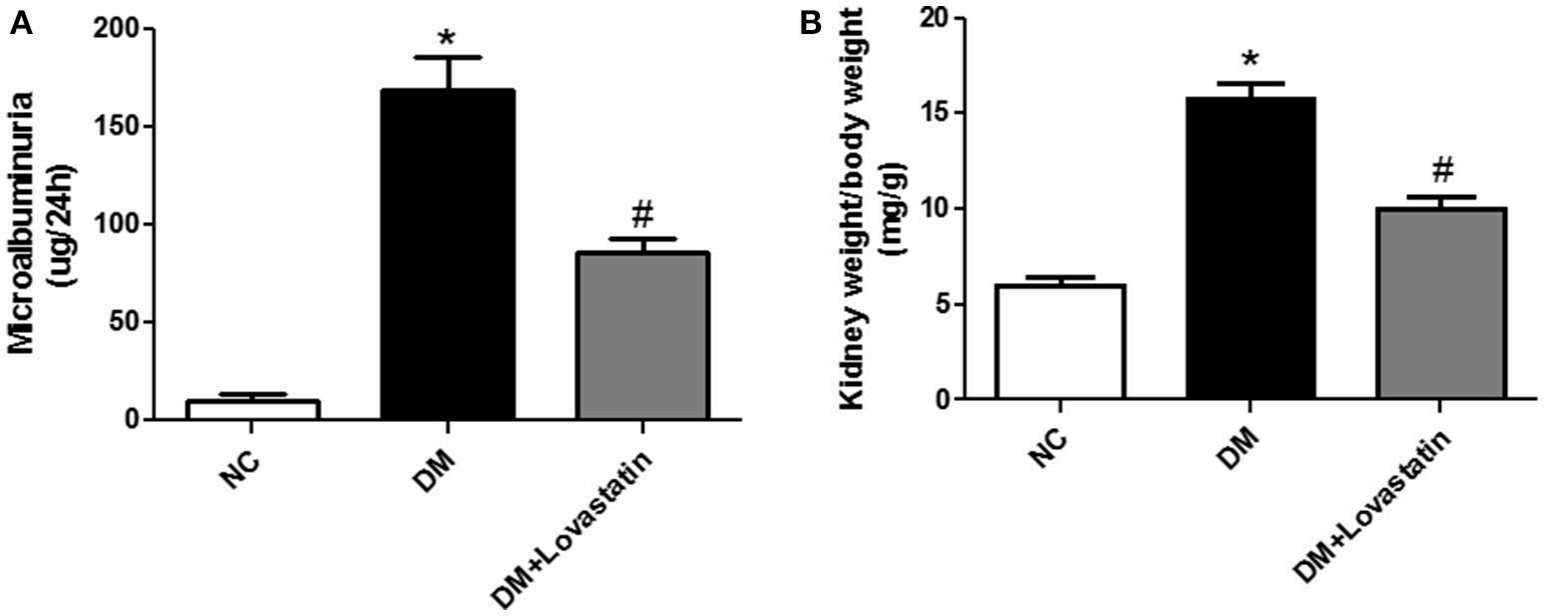
Microalbuminuria and KW/BW. **(A)** Microalbuminuria. **(B)** KW/BW. NC, normal control group; DM, diabetic group; DM +Lovastatin, lovastatin-treated diabetic group. ^*^P < 0.05 between the NC and the DM groups; ^#^*P* < 0.05 between the DM and the DM + Lovastatin groups.

As shown in Table 1, the level of FBG in DM and lovastatin-treated DM group were obviously increased compared with NC group at 16 weeks, (*P* < 0.05), while no change was found between DM group and the lovastatin-treated DM group (*P* > 0.05). There was no significant change of serum TC, TG, Cr, BUN, AST, and AST levels in each group. At the end of the study, microalbuminuria, one of the functional parameters in DN, was dramatically increased in DM rats compared with NC rats, which was reduced by lovastatin administration (*P* < 0.05) (Figure 1A). KW/BW was used to assess glomerular hypertrophy, which was substantially greater in DM rats compared with NC rats (*P* < 0.05), however, KW/BW was decreased in lovastatin-treated DM group (*P* < 0.05), which showed that lovastatin could effectively prevent hypertrophy of kidney (Figure 1B).

**Table 1:** Metabolic parameters of the rats in different groups (Mean ± *SD*).

	**NC (*n* = 10)**	**DM (*n* = 10)**	**DM+lovastatin (*n* = 10)**
Blood glucose (mmol/L)	6.2 ± 0.3	25.3 ± 2.8^*^	25.0 ± 3.2^*^
TC (mmol/L)	2.1 ± 1.4	2.7 ± 1.1	2.4 ± 1.3
TG (mmol/L)	1.3 ± 0.9	1.4 ± 1.0	1.3 ± 0.8
ALT (mmol/L)	49.3 ± 6.6	54.1 ± 5.9	51.8 ± 7.1
AST (mmol/L)	45.3 ± 7.2	47.4 ± 5.7	46.8 ± 6.8
BUN (mmol/L)	5.4 ± 2.2	5.8 ± 1.9	5.7 ± 1.6
Scr (mmol/L)	47.3 ± 6.5	45.9 ± 7.1	49.4 ± 8.2

*TC, total cholesterol; TG, total triglycerides; ALT, alanine transaminase; AST, aspartate transaminase; BUN, blood urea nitrogen; Scr, serum creatinine; NC, normal control group; DM, diabetic group; DM +Lovastatin, lovastatin-treated diabetic group*.

* *p < 0.05 between the CHD and the HFD groups*.

#### Effects of lovastatin on renal fibrosis

**Figure 2:**
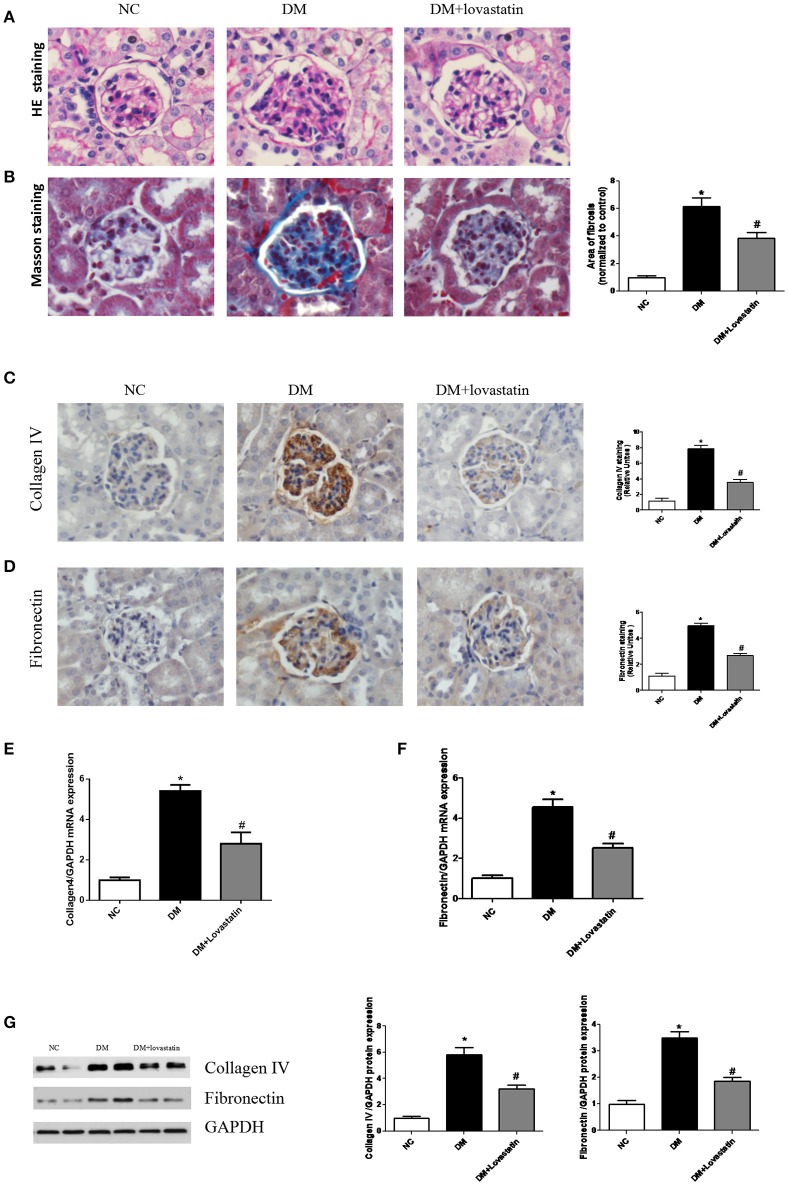
Pathological analysis of rat kidney tissue. **(A)** HE staining. **(B)** Masson's staining. **(C,D)** Immunostaining for Collagen IV and Fibronectin. **(E,F)** The mRNA expression levels of Collagen IV and Fibronectin. **(G)** Western blotting of Collagen IV and Fibronectin proteins in different groups. NC, normal control group; DM, diabetic group; DM +Lovastatin, lovastatin-treated diabetic group. ^*^P < 0.05 between the NC and the DM groups; ^#^*P* < 0.05 between the DM and the DM + Lovastatin groups.

HE staining showed DM rats had increased glomerular volume, thicken the basal membrane, and developed significant mesangial expansion compared with the NC rats, however, treatment with lovastatin reversed these changes to some degree (Figure 2A). Masson staining showed the area of interstitial fibrosis (blue) in the DM rats was markedly increased compared to the NC rats, while it was significantly decreased by lovastatin treatment *P* < 0.01 (Figure 2B).

Immunohistochemistry revealed expressions of pro-fibrotic genes fibronectin and collagen IV in the glomeruli were significantly elevated in DM group comparison with those in NC group, in contrast, treatment with lovastatin mitigated the increased expression of fibronectin and collagen IV (Figures 2C,D). Real-time qPCR assay showed significant increases in mRNA levels of fibronectin and collagen IV (Figures 2E,F). In addition, these observations were further confirmed by western blot analysis (Figure 2G).

#### Effect of lovastatin on EndMT in kidney

**Figure 3:**
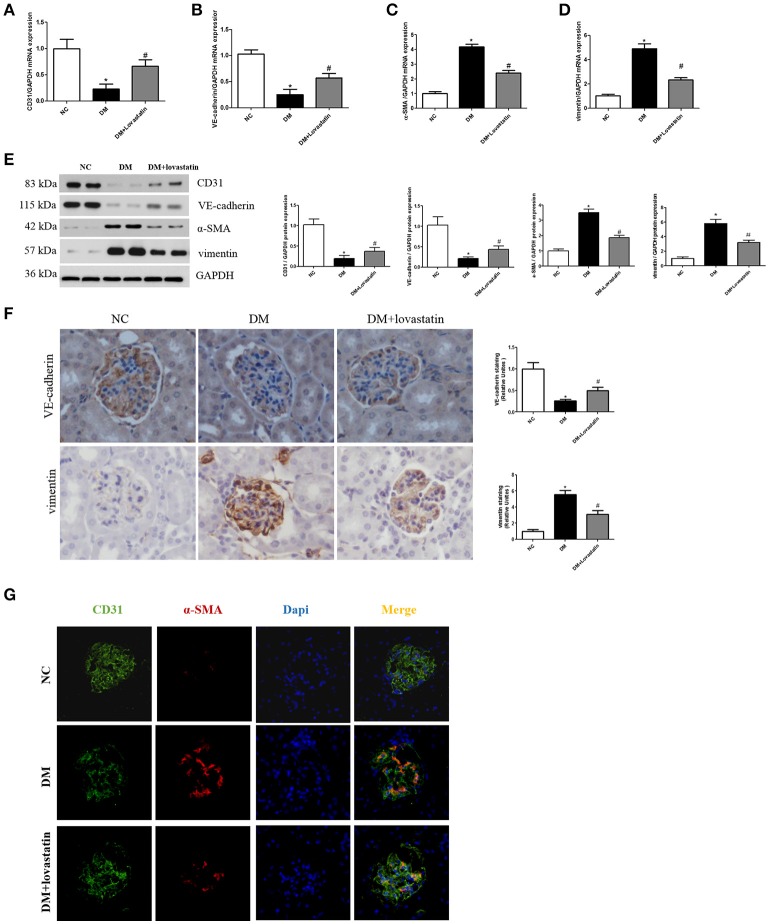
Effect of lovastatin on EndMT in kidney. **(A–D)** The mRNA levels of CD31, VE-cadherin, α-SMA, and vimentin in kidney. **(E)** Western blotting of CD31, VE-cadherin, α-SMA, and vimentin proteins in different groups. **(F)** Immunostaining for VE-cadherin and vimentin. **(G)** Immunofluorescence for CD31 and α-SMA. ^*^P < 0.05 between the NC and the DM groups; ^#^*P* < 0.05 between the DM and the DM + Lovastatin groups.

CD31 and VE-cadherin are endothelial cell marker, α-SMA and vimentin are fibroblast marker. RT-PCR and Western blot analysis showed that both CD31 and VE-cadherin were significantly reduced in DM rats compared with NC rats, while those were partially restored by lovastatin treatment. By contrast, the levels of α-SMA and vimentin were obviously increased in DM rats compared with NC rats, while these expressions were decreased by lovastatin treatment (Figures 3A–E). Immunohistochemistry showed that compared with NC rats, the expression of VE-cadherin in glomeruli was significantly decreased and vimentin was increased in DM group, while these were partly reversed by the treatment with lovastatin (Figure 3F).

Furthermore, we carried out double labeling immunofluorescence to show the colocalization of CD31 and α-SMA. Double positive cells are considered as the evidence of the occurrence of EndMT (Zhang et al., 2016). As shown in Figure 3G, double positive cells were obviously increased in DM group compared to NC group, while the number of double-labeled cells was markedly reduced in lovastatin group. These results indicated that lovastatin could inhibit EndMT in glomeruli.

#### Effects of lovastatin treatment on oxidative stress in diabetic kidney

**Figure 4:**
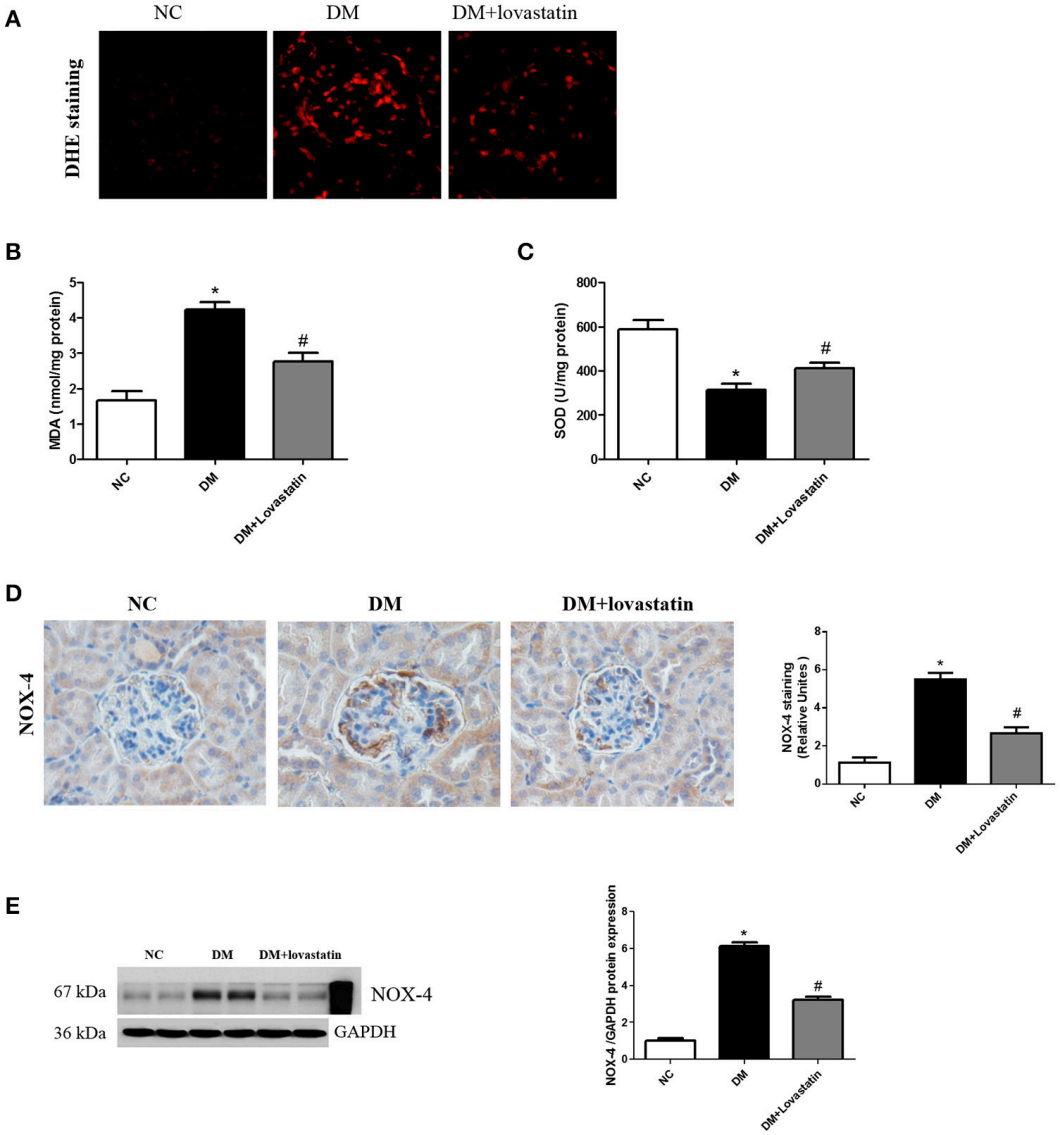
Effect of lovastatin on oxidative stress in kidney. **(A)** DHE staining in kidney sections in each group **(B,C)** The product of lipid peroxidation (MDA) and superoxide dismutase (SOD) in each group. **(D)** Immunostaining for NOX4. **(E)** Western blot of NOX4 proteins in different groups. NC, normal control group; DM, diabetic group; DM +Lovastatin, lovastatin-treated diabetic group. ^*^P < 0.05 between the NC and the DM groups; ^#^*P* < 0.05 between the DM and the DM + Lovastatin groups.

To evaluate the impact of lovastatin on oxidative stress in kidney, DHE staining was carried out to measure the superoxide expression. The results in Figure 4A exhibited that DHE expression was obviously increased in the kidney of DM rats, while lovastatin treatment decreased its expression. In addition, oxidative stress biomarkers including malondialdehyde (MDA) and superoxide dismutase (SOD) were detected. As shown in Figures 4B,C, MDA level in renal tissue was significantly increased in DM rats (*P* < 0.05), which was accompanied by a marked reduction in SOD level (*P* < 0.05); however, these were significantly counteracted in lovastatin treated DM rats. Furthermore, NADPH oxidase isoform (NOX-4) were detected using immunohistochemistry and western blot analysis, DM rats showed increased expression of NOX-4 in kidney compared with NC rats, while treatment with Lovastatin significantly decreased the expression of NOX-4 (Figures 4D–E). These results indicated that lovastatin had a protective effect against oxidative stress in the kidney.

### Lovastatin prevented TGF-β1/Smad activation in diabetic kidney

**Figure 5:**
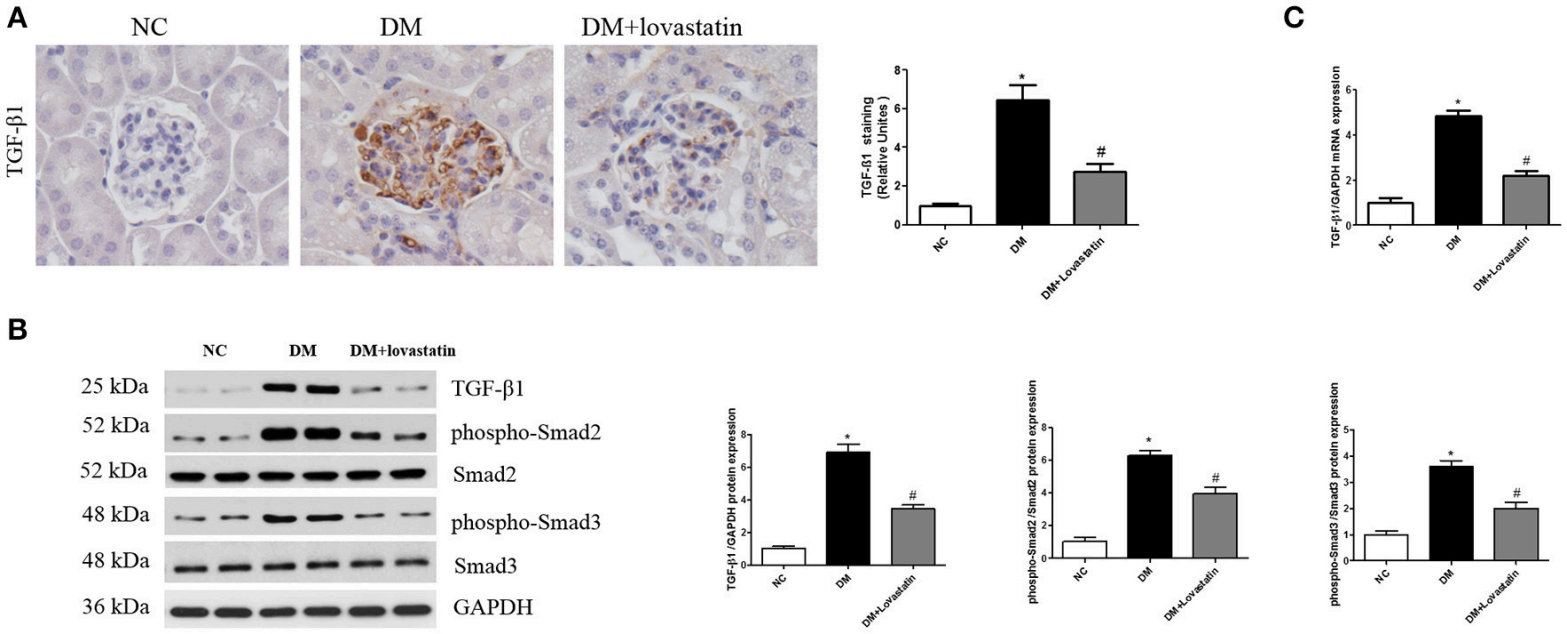
Effect of lovastatin on TGF-β1/Smad in kidney. **(A)** Immunostaining for TGF-β1. **(B)** Western blot of TGF-β1 and phospho-Smad2/3 proteins in different groups. **(C)** The mRNA levels of TGF-β1 in kidney. NC, normal control group; DM, diabetic group; DM +Lovastatin, lovastatin-treated diabetic group. ^*^P < 0.05 between the NC and the DM groups; ^#^*P* < 0.05 between the DM and the DM + Lovastatin groups.

Previous studies have confirmed that TGF-β1/Smad signaling plays a critical role in the develop of EndMT (Wang Z. et al., 2017), therefore, the change of TGF-β1/Smad pathway in the kidney was detected. TGF-β1 immunohistochemistry showed TGF-β1 expression in DM rats was significantly increased compared with NC rats, and lovastatin treatment reduced TGF-β1 expression in diabetic rats (Figure 5A, *P* < 0.05). Western blot results indicated that the level of TGF-β1 and phospho-Smad2/3 in DM group were significantly elevated compared to NC group, while the level of TGF-β1 and phospho-Smad2/3 in lovastatin treated rats were significantly decreased (Figure 5B, *P* < 0.05). Furthermore, we examined mRNA level of TGF-β1 in kidney by RT-PCR (Figure 5C), which was upregulated in the DM group compared to the NC group (*P* < 0.05), while TGF-β1 was obviously downregulated in lovastatin treated group (*P* < 0.05).

### *In vivo* experiments

#### Lovastatin suppress EndMT induced by high glucose in GEnCs

**Figure 6:**
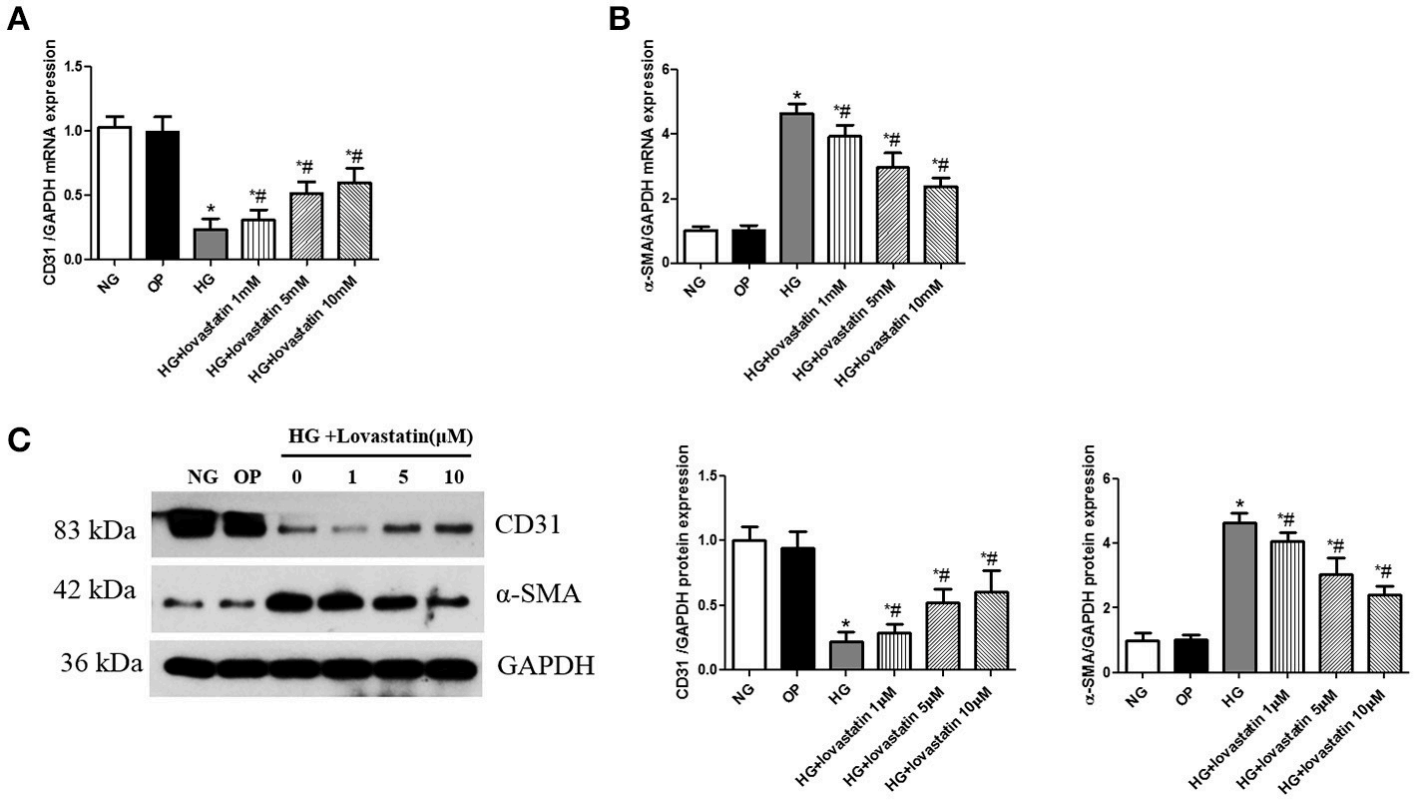
Effect of lovastatin on EndMT induced by high glucose in GEnCs. **(A)** The mRNA levels of CD31 induced by high glucose in GEnCs. **(B)** The mRNA levels of α-SMA induced by high glucose in GEnCs. **(C)** Western blot of CD31 and α-SMA protein induced by high glucose in GEnCs.NG, normal control group; OP, osmotic pressure control group; HG, high glucose group; HG + lovastatin 1 μM, high glucose with lovastatin 1 μM treatment group; HG + lovastatin 5 μM group, high glucose with lovastatin 5 μM treatment group; HG + lovastatin 10 μm, high glucose with lovastatin 10 μM treatment group. ^*^P < 0.05 vs. NG group, ^#^*P* < 0.05 vs. HG group.

To examine if lovastatin could suppress glucose-induced EndMT in GEnCs, we treated GEnCs with lovastatin at 1, 5, or 10 uM before adding 30 mM glucose. As shown in Figures 6A–C, after the cultivation in high glucose for 48 h, mRNA and protein levels of CD31 were decreased, but the expression of α-SMA was increased compared to NG group (*P* < 0.05), in contrast, lovastatin significantly increased the mRNA and protein levels of CD31 and decreased the expression of α-SMA in a dose-dependent manner. These results suggest that lovastatin could suppress high glucose induced EndMT in GEnCs.

#### Lovastatin suppresses HG-induced activation of TGF-β1/Smads signaling in GEnCs

**Figure 7:**
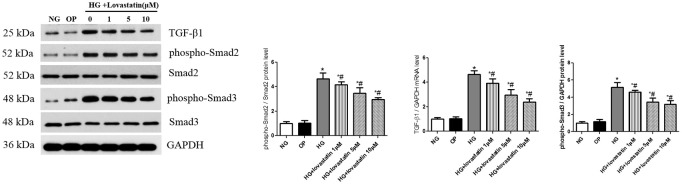
Effect of lovastatin on TGF-β1/Smad signaling induced by high glucose in GEnCs. Western blot of TGF-β1 and phospho-Smad2/3 proteins induced by high glucose in GEnCs. NG, normal control group; OP, osmotic pressure control group; HG, high glucose group; HG + lovastatin 1 μM, high glucose with lovastatin 1 μM treatment group; HG + lovastatin 5 μM group, high glucose with lovastatin 5 μM treatment group; HG + lovastatin 10 μm, high glucose with lovastatin 10 μM treatment group. ^*^P < 0.05 vs. NG group, ^#^*P* < 0.05 vs. HG group.

TGF-β1/Smad is recognized as a vital signaling pathway involved in the process of EndMT induced by HG. Compared with NG group, the protein expression of TGF-β1 and phospho-Smad2/3 were significantly increased after exposing to 30 mmol/L glucose for 48 h (*P* < 0.05), in contrast, in lovastatin + HG group for 48 h, the level of TGF-β1 and phospho-Smad2/3 were significantly decreased in a dose-dependent manner (Figure 7, *P* < 0.05), but there was no change between NG group and OP group. These results indicated that HG-induced activation of TGF-β1/Smads signaling could be suppressed by lovastatin in GEnCs.

## Discussion

DN is a serious microvascular complication of DM (Ahmad, 2015), accounting for the cardiovascular complications and high mortality rate of patients with diabetes. DN is characterized by glomerular hypertrophy, proteinuria, podocyte, and tubular cell injury, reduced glomerular filtration, glomerulosclerosis and tubulointerstitial fibrosis. Microalbuminuria is regarded as a characteristic of DN and shows injury of the glomerular filtration barrier which comprises GECs, podocytes and the glomerular basement membrane (GBM) (Gnudi et al., 2016). Previous studies mainly focused on the podocytes, however, increasing evidence suggests that dysfunction of glomerular endothelial cells also plays a key role (Quaggin and Kreidberg, 2008; Scott and Quaggin, 2015).

Studies have shown statins, HMG-CoA reductase inhibitors, have renal protective effects by inhibiting HG-caused ROCK1 activation (Peng et al., 2013), however, the underlying molecular mechanism of its renal protective effect is still remain unclear. In the present study, STZ induced diabetes are used as a model for research on DN and showed that lovastatin could reduce microalbuminuria and renal hypertrophy, inhibit EndMT and prevent renal fibrosis without affecting blood glucose and blood lipids level. These effects are involved in reduced ROS generation and inhibition of TGF-β1/Smad. This is the first report demonstrating that lovastatin could alleviate hyperglycemic-induced renal fibrosis via inhibition of EndMT.

Glomerular and tubular interstitial fibrosis are regarded as the main characteristic of DN. Evidence suggests that the epithelial-to-mesenchymal transition (EMT) is a complex reprogramming of cell phenotype in which epithelial cells acquire a mesenchymal phenotype and plays pivotal roles in renal dysfunction and fibrosis (Liang et al., 2016). In the process of renal fibrosis, EMT is regarded as a direct contributor to produce excessive extracellular matrix. GEnCs as a special type of epithelial cells share many common traits with epithelial cells and can undergo similar EMT process, called EndMT. Recent studies suggested that EndMT has emerged as a novel pathway involved in the developmental and pathological processes of tissue fibrosis, including renal fibrosis (Sun et al., 2016). In addition, EndMT appears to play an important role in diabetic renal fibrosis (Li and Bertram, 2010). EndMT is a process by which endothelial cells progressively lose their intercellular adhesion complexes such as VE-cadherin and CD31, and acquire mesenchymal fibroblast-like markers including vimentin and α-SMA. In the present study, CD31 and VE-cadherin were used as the endothelial marker and α-SMA and vimentin as the mesenchymal marker. Our results showed that compared with NC group, the levels of CD31 and VE-cadherin were decreased, while α-SMA and vimentin were increased in DM group, those were markedly reversed partly by lovastatin, suggesting lovastatin could alleviate hyperglycemic-induced EndMT, our *vitro* experiments also indicated that lovastatin could suppress high glucose induced EndMT.

Numerous studies show that EndMT is regulated by some bioactive molecules, such as TGF-β, angiotensin II (AngII) and so on (Perez et al., 2017). In recent years, accumulating evidence suggests TGF-β/Smad signaling is recognized as a crucial factor involved in the process of EndMT (Kumarswamy et al., 2012; Cooley et al., 2014). Inhibition of TGF-β1 can obviously inhibit the EndMT induced by high glucose. Our study indicated the level of collagen and fibronectin were upregulated along with the increase levels of TGF-β1 and p-Smad2/3 in DN, while, lovastatin effectively reversed these changes in diabetic rats. And also, our *vitro* study indicated that HG-induced activation of TGF-β1/Smads could be suppressed effectively by lovastatin in GEnCs.

Reactive oxygen species exerts an important effect in the development of DN (Fernandes et al., 2016). Chronic hyperglycemia-induced oxidative stress is considered the most important factor in TGF-β1 production, which can lead to renal injury and interstitial fibrosis (Forbes et al., 2008). MDA is the end-product of lipid peroxidation and is considered as a marker of oxidative stress (Popov et al., 2003). Our data showed that the production of ROS and MDA were increased in the DM-induced diabetic group, which was accompanied by the impairment of SOD activity, while lovastatin considerably reduced the generation of ROS and MDA, and also it up-regulated the activity of SOD, in addition, lovastatin decreased the expression of NOX4 in the DM-induced diabetic group. These findings corroborate those of earlier studies demonstrating that statins could significantly attenuate renal damage and prevent endothelial dysfunction by reducing oxidative stress (Li and Bertram, 2010; Zinellu et al., 2015; Wang F. et al., 2017).

In summary, our data demonstrated that lovastatin could attenuate renal damage, ameliorate EndMT in Glomeruli, the mechanism of which may be at least partly through suppression of oxidative stress and TGF- β1/Smad signaling pathway.

## Ethics statement

All animal studies were approved by the Animal Care and Use Committee of Tianjin Medical University, and were carried out in accordance with the Guide for the Care and Use of Laboratory Animal published by the US National Institutes of Health and Tianjin Medical University.

## Author contributions

ZM, LZ, LC, and QL conceived and designed the experiments; ZM, LZ, YY, YL, and ZW performed the experiments; ZM, YY, LC, and ZW analyzed the data; ZM and QL wrote the paper.

### Conflict of interest statement

The authors declare that the research was conducted in the absence of any commercial or financial relationships that could be construed as a potential conflict of interest.

## Abbreviations

AngII: angiotensin II
α-SMA: α-smooth muscle actin
CKDs: chronic kidney diseases
DAB: Diaminobenzidine
CTGF: connective tissue growth factor
DN: Diabetic nephropathy
ECM: endothelial cell medium
ECs: endothelial cells
EndMT: endothelial-to-mesenchymal transition
ESRD: end-stage renal disease
FSP-1: fibroblastspecific protein 1
GEnCs: glomerular endothelial cells
HMG-CoA: 3-hydroxy-3-methylglutaryl-coenzyme A
KW/BW: kidney weight to body weight
MDA: malondialdehyde
NOX-4: NADPH oxidase isoform
PDGF: platelet-derived growth factor
ROS: Reactive oxygen species
SOD: superoxide dismutase
TGF-β1: transforming growth factor-β1
